# Adjunct Automated Breast Ultrasound in Mammographic Screening: A Systematic Review and Meta-Analysis

**DOI:** 10.3390/jimaging12010003

**Published:** 2025-12-22

**Authors:** Ghufran Jassim, Fahad AlZayani, Suchita Dsilva

**Affiliations:** 1 Department of Family Medicine, School of Medicine, Royal College of Surgeons in Ireland-Medical University of Bahrain, Busaiteen 15503, Bahrain; sdsilva@rcsi-mub.com; 2 School of Medicine, Arabian Gulf University, Manama 22979, Bahrain; fahadrkz@agu.edu.bh

**Keywords:** breast density, screening, automated breast ultrasound, handheld ultrasound, mammography, diagnostic accuracy

## Abstract

Mammographic sensitivity is reduced in women with dense breasts, leading to missed cancers and a higher burden of interval cancers. Automated breast ultrasound (ABUS) and ultrasound tomography (UST) have been introduced as supplemental breast imaging modalities, but primary studies are heterogeneous, and previous reviews have not focused on screening settings or on head-to-head comparisons with handheld ultrasound (HHUS). We systematically searched PubMed, Embase, Web of Science and the Cochrane Library for studies from 1 January 2000 to 31 May 2025 evaluating ABUS or UST as adjuncts to mammographic screening. Two reviewers independently selected studies and assessed risk of bias. When at least two clinically comparable studies were available, we pooled sensitivity and specificity using random-effects bivariate meta-analysis. Eighteen studies (just over 20,000 screening or recall episodes) met the inclusion criteria; 16 evaluated ABUS/ABVS and 2 UST. Adding ABUS to mammography increased sensitivity by 6–35 percentage points and improved cancer detection by 2.4–4.3 per 1000 women with dense breasts, with higher recall rates and modest reductions in specificity. When ABUS was compared directly with HHUS, pooled sensitivity was 0.90 and specificity 0.89, with HHUS showing slightly lower sensitivity and slightly higher specificity. Only two studies had an overall low risk of bias, and heterogeneity (particularly for specificity) was substantial. ABUS is a practical and scalable adjunct to mammography that increases cancer detection in women with dense breasts, with an expected trade-off of higher recall and modest specificity loss. Its comparative diagnostic accuracy appears broadly non-inferior to HHUS. However, the predominance of high-risk-of-bias studies and between-study heterogeneity means that high-quality population-based trials and standardised reporting are still required before widespread implementation in organised screening programmes.

## 1. Introduction

Worldwide, female breast cancer is the most prevalent cancer, with an estimated 2.3 million new cases and 685,000 deaths in 2020 [1]. Mammography is the standard screening modality and can achieve high sensitivity in average-density breasts [2], but performance falls markedly in women with heterogeneously or extremely dense breasts, where fibroglandular tissue can mask lesions and lead to misdiagnosis and a higher proportion of interval cancers [3,4]. Breast density decreases with age but remains high in up to half of women in screening programmes [3,4] and is itself an independent risk factor for breast cancer [5]. The fifth edition of the Breast Imaging Reporting and Data System (BI-RADS) classifies density qualitatively into four categories (A–D); women with BI-RADS C/D (“heterogeneously” or “extremely” dense) experience both reduced mammographic sensitivity and increased breast cancer risk [6].

However, in most countries, women with an average breast cancer risk, including those with dense breast tissue, are currently screened using mammography [2]. Therefore, it has been recently advocated that screening with ultrasound as an adjunct to mammography in dense breasts is as beneficial as contrast-enhanced MRI in extremely dense breasts [7].

The standard supplemental screening technique in women with dense breasts is hand-held whole-breast ultrasound (HHUS), but this approach is operator-dependent and time-consuming. Automated breast ultrasound (ABUS), including automated breast volume scanners (ABVS), was approved by the US Food and Drug Administration (FDA) in 2012 and provides standardised volumetric coverage of the whole breast, with technologist-acquired 3-D sweeps reviewed by radiologists at a workstation [8,9]. Ultrasound tomography (UST) is a newer three-dimensional ultrasound technique that acquires tomograms of sound speed and related acoustic properties using a 360-degree transducer array in a water bath [10]. Because it uses sound waves rather than ionising radiation, UST can image dense breasts without radiation exposure; the first clinical UST system received FDA clearance in 2021 [10].

ABUS is a volumetric sonographic technique in which the volume of the entire breast volume is acquired [11]. The main advantage of ABUS over HHUS is the standardised acquisition, with a decrease in both operator dependency and physician workload [11]. UST is performed with a proprietary 360-degree ring transducer, scanning each breast from the chest wall to the nipple while the patient is lying prone and her breast is submerged in a warm water bath [12]. The benefit of this technique is the short time required to perform the test (between 2 and 4 min for each breast), its privacy as the breast is not visible during the scanning process, operator independence, and it is less painful as there is no breast compressing involved [12].

Many studies including a systematic review have compared the performance of ABUS and HHBUS and have shown comparable and promising findings in the screening setting, especially in women with dense breasts [13,14,15,16].

### Relevance and Rationale of This Review

There is growing interest in adjunct imaging for women with dense breasts, and several automated ultrasound platforms are now commercially available.

Despite these potential benefits, adjunct ultrasound is not routinely integrated into most population-based mammographic screening programmes. Adoption of handheld ultrasound and ABUS is largely confined to selected high-risk clinics, recall assessments or regional pilots, while only a few organised screening programmes systematically invite women with dense breasts for supplemental ultrasound. Barriers include increased recall rates and downstream biopsies, limited radiologist capacity, additional equipment and training costs, uncertainty about effects on interval cancer rates and mortality, and heterogeneous evidence from predominantly single-centre or enriched cohorts [17]. Against this backdrop, it is important to clarify whether newer automated ultrasound technologies offer sufficient diagnostic and operational advantages over HHUS and mammography alone to justify broader implementation.

To address these gaps, we undertook a systematic review and meta-analysis focused on synthesising the evidence regarding automated ultrasound technologies used in screening and screening-recall contexts, with standardised quality assessment and formal synthesis of both diagnostic accuracy and key programme-level outcomes.

## 2. Materials and Methods

**Figure 1:**
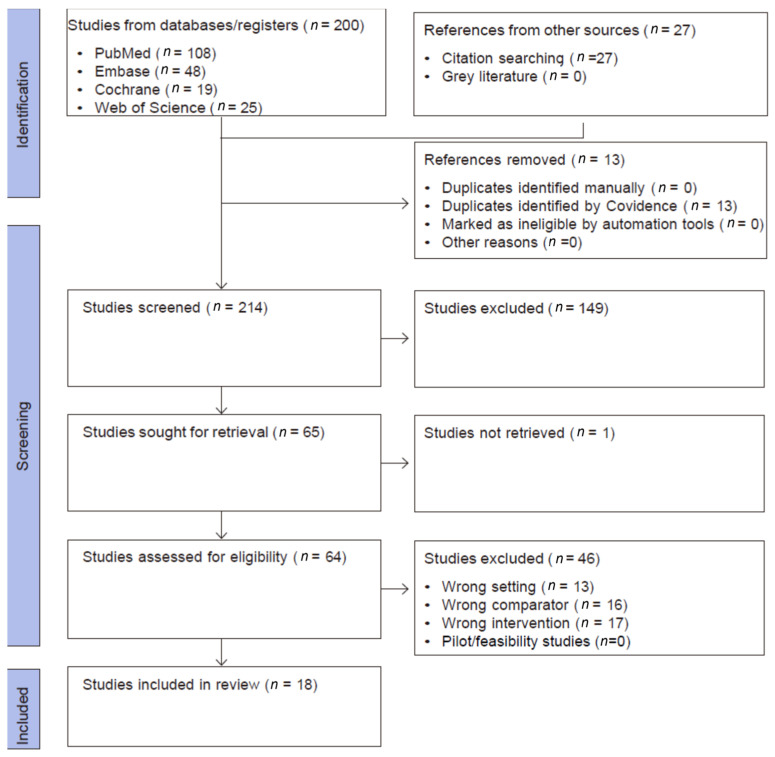
PRISMA flow diagram.

This systematic review and meta-analysis was conducted and reported in accordance with the PRISMA (Preferred Reporting Items for Systematic Reviews and Meta-Analyses) 2020 statement. All stages of the review process-including study selection, data extraction, risk of bias assessment, and synthesis followed PRISMA guidelines to ensure transparency, reproducibility, and methodological rigour. A PRISMA flow diagram was used to illustrate the study selection process (Figure 1). The review is registered with inplasy.com; registration number is INPLASY2025100111 and DOI number is 10.37766/inplasy2025.10.0111.

### 2.1. Search Methods for Identification of Studies

A comprehensive search strategy was implemented in PubMed, Embase, Web of Science Core Collection, and the Cochrane Library from 1 January 2000 to 31 May 2025. The lower date limit was chosen to precede the introduction of ABUS by approximately a decade and to capture early studies of automated ultrasound. No language restrictions were applied. The overall flow of records through the identification and screening process is summarised in Figure 1.

The search combined controlled vocabulary (MeSH/Emtree terms) and free-text keywords across four conceptual domains: breast neoplasms, mass screening, ultrasound modalities (ABUS, handheld ultrasound, and ultrasonography), and mammography, with breast density as a modifying factor. Boolean operators (“AND”, “OR”) were employed to optimise sensitivity. An illustrative PubMed search string was used as follows:

1. (“Breast Neoplasms”[MeSH Terms] OR “Breast Neoplasms”[Title/Abstract] OR “breast cancer” OR “breast carcinoma”) AND (“Mass Screening”[MeSH Terms] OR “Mass Screening”[Title/Abstract] OR “screening” OR “detection”) AND (“Ultrasonography”[MeSH Terms] OR “automated ultrasound” OR “ABUS” OR “handheld ultrasound” OR “ultrasound” OR “automated breast ultrasound”) AND (“Mammography”[MeSH Terms] OR “Mammography”[Title/Abstract] OR “mammogram” OR “mammographic”) AND (“Breast Density”[MeSH Terms] OR “breast density”).

To reduce the risk of publication bias and missed studies, we supplemented database searches with backward and forward citation tracking of all included articles and key reviews, using citation tools embedded in PubMed, Embase, and Web of Science. We also screened grey-literature sources (conference abstracts and trial registries) to identify additional or ongoing studies. Multiple reports from the same study were collated and treated as a single unit of analysis.

### 2.2. Eligibility Criteria for Study Selection

We included primary studies evaluating the adjunct use of ABUS, ABVS, or UST in breast cancer screening compared to mammography alone, mammography + HHUS, or HHUS alone. Key outcomes included sensitivity/specificity, cancer detection rate (CDR), recall, predictive values, and workflow metrics. Eligible designs included prospective or retrospective diagnostic-accuracy studies and screening cohorts; randomised/non-randomised controlled or non-controlled; conducted in a screening (population-based or opportunistic) or recall/work-up context. Any form of mammography screening (e.g., one view, two views, digital, tomosynthesis (three-dimensional (3D)-mammography), combination of 2D- and 3D-mammography) was included. The exclusion criteria were applied in two distinct stages. During the title and abstract screening, studies were excluded for reasons such as duplication. In the full-text screening stage, additional exclusions were made for wrong population, wrong index test, wrong setting, or wrong comparator, pilot or feasibility designs due to small sample sizes (fewer than 20 participants) and for studies lacking relevant test-accuracy or screening-performance outcomes. The flow of study selection is summarised in Figure 1.

Two authors independently reviewed the titles and abstracts of selected articles. We retrieved the full text of all the studies that met the inclusion criteria. We also searched for references to these studies and to systematic reviews. Disagreements were resolved through discussion, and if needed, the third author resolved any remaining disputes.

We pilot-tested the eligibility criteria on a sample of reports (six articles, including those that were thought to be definitely eligible, definitely not eligible, and doubtful). A pilot test was used to refine and clarify the eligibility criteria and ensure consistency between the investigators. Coevidence software was used to automate the selection process (www.covidence.org (assessed on 11 May 2025)).

As shown in Figure 1, the searches yielded 200 eligible articles, and 27 articles were included from the references of the relevant studies. Thirteen duplicates were excluded, and 214 titles were screened for eligibility. The full texts of 64 articles were retrieved. Two non-English studies required professional translation. The selection process is illustrated in Figure 1.

### 2.3. Data Extraction and Management

Structured data-extraction forms were designed and used to gather pertinent information from the included articles. This includes the characteristics of the study population, settings, index tests, comparators, study designs, and outcomes.

### 2.4. Assessment of Risk of Bias in Included Studies

Two authors independently assessed the risk of bias using QUADAS-2 the (Quality Assessment of Diagnostic Studies) tool, which is specifically designed to evaluate diagnostic accuracy studies [18]. This tool evaluates four key domains: 1. patient selection; 2. index test; 3. reference standards; and 4. flow and timing. The first three domains were assessed for applicability. For each domain, the reviewers provided a judgement of the risk of bias (low, high, or unclear) accompanied by justification based on the information reported in the study. Any disagreements between reviewers were resolved through discussion.

When the required figures were missing, they were derived from the text, tables, or flow diagrams. If derivation was impossible, the study was narratively synthesised and excluded from the relevant meta-analysis.

We assessed the certainty of the evidence for each primary outcome using the GRADE approach for diagnostic tests, considering risk of bias (QUADAS-2 domains), inconsistency, indirectness, imprecision, and potential publication bias. For each outcome, we started at high certainty and downgraded as appropriate based on prespecified criteria, and then classified the overall certainty as high, moderate, low, or very low.

### 2.5. Data Synthesis and Analysis

We summarised the characteristics of included studies narratively and extracted or reconstructed 2 × 2 tables (true positives, false positives, false negatives, true negatives) for each eligible comparison. When studies reported only percentages, we back-calculated counts from the reported denominators where possible. For zero cells in the 2 × 2 tables, a continuity correction of 0.5 was applied. When multiple readers were reported for the same dataset, we used the consensus read, or if not available, the primary analysis read to avoid unit-of-analysis errors.

For quantitative synthesis of diagnostic accuracy, we used a bivariate random-effects model with hierarchical summary receiver-operating-characteristic (HSROC) parameterisation, jointly modelling logit-transformed sensitivity and specificity and their between-study covariance. This approach accounts for between-study heterogeneity and the potential correlation between sensitivity and specificity. We present summary points with 95% confidence regions, alongside forest plots showing study-specific and pooled estimates with 95% confidence intervals. Meta-analysis was performed only when at least two clinically comparable studies contributed data to a given comparison.

### 2.6. Assessment of Heterogeneity and Publication Bias

Between-study heterogeneity was quantified using Cochran’s Q statistic and the I^2^ statistic, which describes the percentage of total variability in effect estimates that is due to heterogeneity rather than chance. I^2^ values of approximately 25%, 50% and 75% were interpreted as low, moderate and high heterogeneity, respectively [19]. Pooled estimates of sensitivity and specificity were obtained using random–effects models to account for between-study variability.

Potential publication bias and small–study effects were explored visually by inspection of funnel plots of logit-transformed effect size against its standard error, and formally tested using Egger’s regression asymmetry test, with a two–sided *p* value < 0.05 considered indicative of statistically significant asymmetry.

## 3. Results

### 3.1. Characteristics of Included Studies

**Figure 2:**
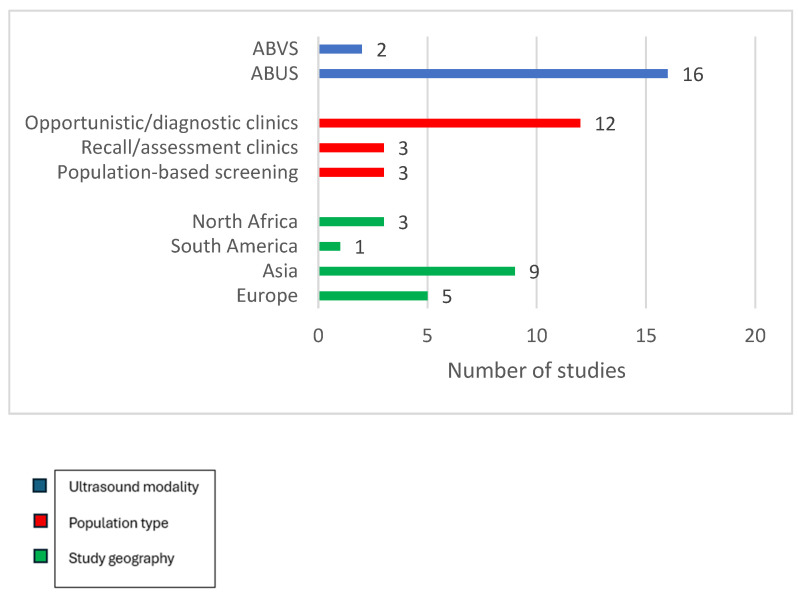
Key characteristics of included studies.

We included 18 studies with sample sizes ranging from a pilot study of 25 Egyptian participants [20] to a Korean cohort of 5566 women [21], with a combined population of just over 20,000 screening or recall episodes. Fifteen studies were prospective, three were retrospective, and nine were multicentre studies. All but two were conducted in hospital-based secondary or tertiary services; three [15,22,23] embedded ABUS into population screening workflows, whereas the remainder evaluated ABUS or ABVSin opportunistic screening, recall assessment or breast clinics. Nine studies included only a cohort of women with dense breasts, defined as BI-RADS C/D or ACR 3/4 women [14,15,20,22,24,25,26,27,28]. Sixteen studies employed ABUS as the index text and only two used ABVS [21,28]. The geographical coverage was broad in Europe (Italy, the Netherlands, Poland, and Sweden), Asia (China, Korea, Iraq, and Turkey), South America (Brazil), and North Africa (Egypt). Figure 2 illustrates the key characteristics of the included studies.

Consecutive recruitment predominated, minimising selection bias, although two studies used random or enriched sampling to ensure adequate numbers of dense breasts or lesion-positive cases. Additional information detailing the designs and contexts of the 18 included studies is presented in the Supplementary Materials Tables S1 and S2. 

All studies acquired at least three automated 3-D sweeps per breast (anteroposterior, lateral, and medial), reconstructed in axial, sagittal, and coronal planes; most used the GE Invenia™ platform (GE healthcare, Chicago, IL, USA), while four used Siemens Acuson S2000 (Siemens Healthineers, Erlangen, Germany), and one used the prone Sofia™ system [22]. HHUS comparators were performed with high-frequency (5–18 MHz) linear probes, usually in a radial/antiradial fashion, and interpreted blindly to ABUS. Histopathology was the dominant reference standard, with core needle biopsy for all BI-RADS 4/5 lesions supplemented by follow-up imaging for BI-RADS 1–3 cases. Reader expertise varied from trainees with ≥100 ABUS teaching cases to senior breast radiologists. Four studies reported substantial inter-reader agreement for ABUS, ranging from (κ 0.57–0.85) [22,24,26,29]. Reported workstation reading times were short, between 3 and 5 min per bilateral study, while technologist acquisition times ranged from 3 min (supine Invenia) to 35 s (single-pass prone Sofia), highlighting the potential to redistribute workload away from the radiologists.

### 3.2. Assessment of Risk of Bias

**Figure 3:**
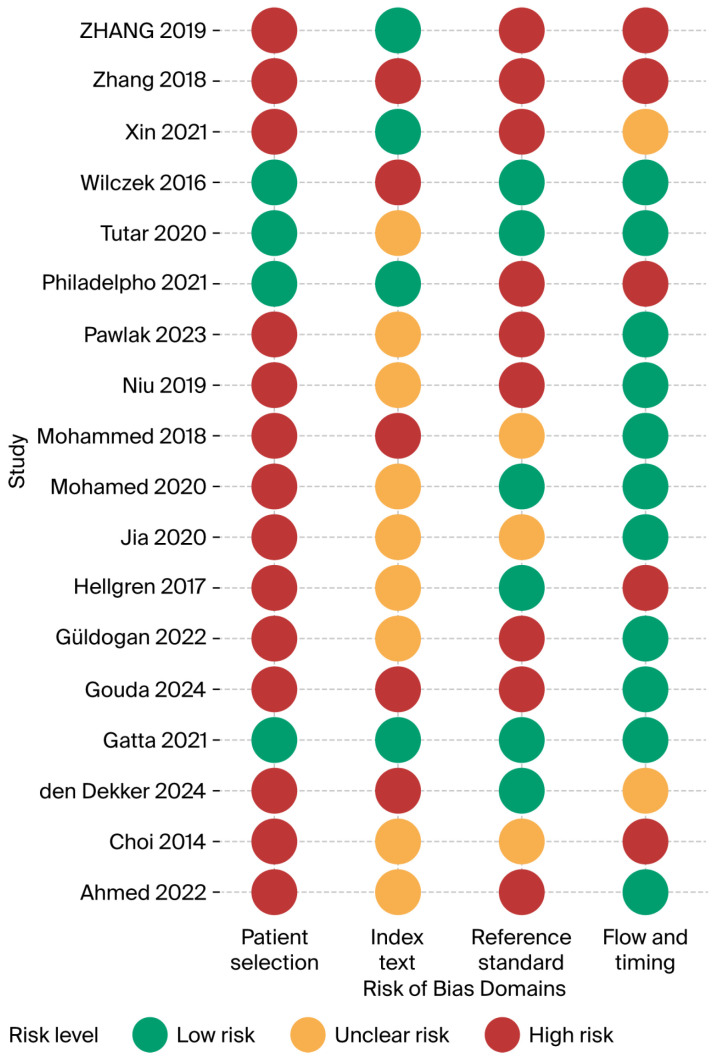
Risk of bias summary (Ahmed 2022 [24], Choi 2014 [21], den Dekker 2024 [30], Gatta 2021 [22], Gouda 2024 [25], Güldogan 2022 [29], Hellgren 2017 [23], Jia 2020 [26], Mohamed 2020 [27], Mohammed 2018 [20], Niu 2019 [31], Pawlak 2023 [32], Philadelpho 2021 [14], Tutar 2020 [28], Wilczek 2016 [15], Xin 2021 [33], Zhang 2018 [34], Zhang 2019 [35]).

The 18 included studies were assessed in each of the four QUADAS-2 domains and showed a clear, recurring pattern of methodological weaknesses, concentrated in patient selection and verification methods, whereas flow/timing was generally handled well. Of the 18 included studies, 13 scored high risk in at least two domains, three scored low risk in at least three domains and will be used in the sensitivity analysis [15,22,28]. Detailed justification of the scoring is presented in the Supplementary Materials Table S3 and outlined in Figure 3.

#### 3.2.1. Patient-Selection Bias

A total of 14 of 18 studies (78%) were judged high risk in this domain. Most enrolled convenience samples of asymptomatic or clinically referred women or applied restrictive inclusion criteria that excluded many eligible screens; for example, Zhang et al. excluded 871/2 844 women after enrolment [34]. Only four screening cohorts [14,15,22,28] succeeded in recruiting consecutive dense breast invitees, yielding a low-risk judgement. Spectrum bias likely inflates accuracy estimates and limits the generalisability of population screening.

#### 3.2.2. Index-Test Bias

Reader blinding has often been poorly described. Eight studies (44%) were rated unclear because ABUS reviewers may have seen HHUS or mammography. Five studies (28%) were high-risk, notably [30] where the same radiologist added ABUS after reviewing DBT + HHUS, and [15] where ABUS was read immediately after FFDSM. Only five studies reported rigorous, independent, blinded interpretation [14,22,28,34,36]. A lack of blinding means that the index test specificity may have been overestimated in several reports.

#### 3.2.3. Reference-Standard Bias

Half of the studies (9/18) were high-risk because negatives were verified only by short follow-up imaging. Three more studies were unclear owing to heterogeneous follow-up [20,21,26]. Robust pathology for BI-RADS 4/5 plus ≥18-month registry follow-up lowered the risk in six studies.

#### 3.2.4. Flow and Timing

Ten studies (56%) achieved low risk; index tests were performed the same day, and there were uniform verification pathways. Five (28%) were high risk, mainly because of differential follow-up or large post-enrolment exclusions. The condition of the remaining three patients was unclear.

### 3.3. Diagnostic Performance Outcomes

Across studies that compared the combination of mammography with ABUS with mammography alone, sensitivity improved consistently by 6–35 percentage points, and the cancer-detection rate (CDR) increased by 2.4–4.3 per 1000 women. In a large multicentre Chinese series of 937 dense-breast women, mammography + ABUS achieved 99.1% sensitivity and 86.9% specificity (AUC 0.93), essentially matching mammography + HHUS, while offering a modest specificity advantage [26]. Gatta 2021 found sensitivity increased from 58.8% with full-field digital mammography (FFDM) alone to 93.5% when prone ABUS was added, at the cost of a decrease in specificity from 94% to 87% and an absolute recall increase of 12.1/1000 screens [22]. A similar pattern was seen in Sweden: Wilczek et al. reported a CDR gain of 2.4/1000 and a recall rise of 9.0/1000 when ABUS supplemented biennial FFDM, with little change in PPV-recall (30% vs. 29%) [15].

The results were less uniform when ABUS was pitted directly against HHUS. In the multinational non-inferiority trials [33,34] ABUS achieved non-inferior sensitivity (92%) and higher specificity (93% vs. 89%) relative to HHUS, yielding similar AUCs (0.92 vs. 0.90) and excellent κ agreement (0.85). Smaller single-centre comparisons were mixed: Niu 2019 showed higher sensitivity but slightly lower specificity for ABUS (92%/78%) versus HHUS (82%/80%) with an AUC advantage (0.85 vs. 0.81, *p* = 0.041) [31], whereas Güldogan 2022 reported identical sensitivity (100%) but with a specificity penalty for ABUS (82% vs. 91%) and twice the recall rate (19% vs. 10%) [29]. The agreement for lesion categorisation between modalities was generally substantial (κ 0.61–0.79).

The predictive values reflect these trade-offs: In dense breast screening, the addition of ABUS frequently lowered PPV1 because of more recalls (e.g., PPV1 fell from 68% to 25% in Gatta 2021) but maintained or improved PPV for biopsy (PPV3) when targeted biopsy triage was used. Negative predictive value was uniformly high (>92%) for all ABUS comparisons, underpinning its safety as a rule-out test; the Jia 2020 dense-breast cohort recorded an NPV of 99.7% for both ABUS and HHUS in mammography-negative women [26].

Workflow measures favoured automation. In the Brazilian screening clinic ABUS reading averaged 4 min 25 s compared with 5 min 03 s for HHUS, despite equivalent cancer yield and 80.9% overall concordance [14]. Technologist-only acquisition in all but one study eliminated radiologist scanning time, and the single-pass prone system demonstrated sub-minute acquisition per breast, suggesting capacity gains for high-volume programmes.

In summary, evidence from diverse clinical settings indicates that ABUS, when added to mammography or used instead of HHUS as the supplemental ultrasound modality, consistently increases cancer detection in women with dense breasts, at the expense of higher recall rates and modest reductions in specificity. Its high NPV, rapid technologist-driven acquisition, and short reading times support its feasibility for large-scale implementation, although the variability in PPV and specificity points to a learning curve, and the need for reader training and quality assurance.

**Figure 4:**
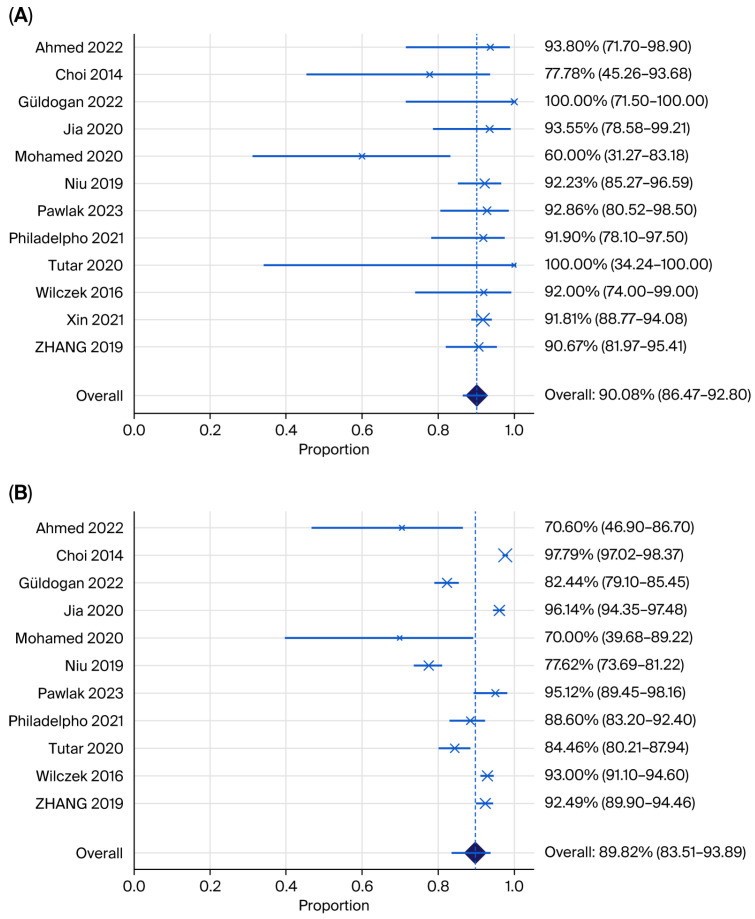
Forest plots showing (**A**) pooled sensitivity and (**B**) pooled specificity for ABUS plus mammography (Ahmed 2022 [24], Choi 2014 [21], Güldogan 2022 [29], Jia 2022 [26], Mohamed 2020 [37], Niu 2019 [31], Pawlak 2023 [32], Philadelpho 2021 [14], Tutar 2020 [28], Wilczek 2016 [15], Xin 2021 [33], Zhang 2019 [35]).

**Figure 5:**
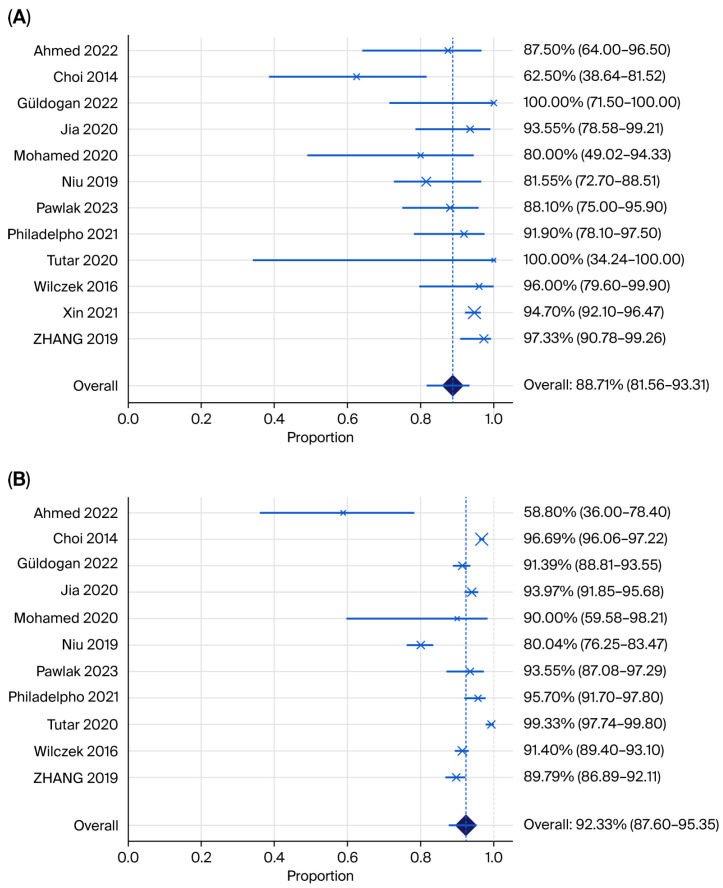
Forest plot showing (**A**) pooled sensitivity and (**B**) specificity for HHUS plus mammography (Ahmed 2022 [24], Choi 2014 [21], Güldogan 2022 [29], Jia 2022 [26], Mohamed 2020 [37], Niu 2019 [31], Pawlak 2023 [32], Philadelpho 2021 [14], Tutar 2020 [28], Wilczek 2016 [15], Xin 2021 [33], Zhang 2019 [35]).

Pooled estimates and illustrative forest plots (Figure 4 and Figure 5) for the sensitivity and specificity of automated breast ultrasound (ABUS/ABVS) when used as the primary index test. Twelve eligible studies reported 2 × 2 data points required for pooled sensitivity and 11 for specificity.

The pooled sensitivity of ABUS across 12 studies was 90.08% (95% CI 86.47–92.80%; I^2^ = 23.97%) with low between-study heterogeneity. Most point estimates were clustered above 0.88, and three studies reported perfect or near-perfect cancer detection.

The pooled specificity of ABUS was 88.71% (95% CI 80.1–91.4; I^2^ = 96.22%), with high heterogeneity. Specificity varied widely from 70% in the small Iraqi series to over 93% in the Chinese multicentre cohort, reflecting reader experience and study purpose (screening versus recall assessment) [24,26]. Tutar et al. [28] evaluated ABVS among MG-negative women only, which inflates recall and makes the specificity not directly comparable to full-cohort designs.

The pooled sensitivity of HHUS across 11 studies was 88.71% (95% CI 81.56–93.31; I^2^ = 70.56%), with high heterogeneity, which was lower than the pooled sensitivity of ABUS. The pooled specificity of HHUS was 92.33% (95% CI 87.60–95.35; I^2^ = 95.71%), with substantial heterogeneity, which was higher than the pooled rate for ABUS.

Across the included studies, recall rates consistently increased when ABUS was incorporated into screening pathways. Three population-level screening studies reported absolute recall increases of 7–12 per 1000 screens when ABUS was added to mammography; however, these higher recall rates were accompanied by meaningful gains in cancer detection, which increased by 2.4–3.4 per 1000 examinations. Positive predictive value (PPV) for biopsy generally improved when ABUS replaced hand-held ultrasound (HHUS), increasing from 84% to 92% in one study. In contrast, when ABUS was added to mammography, PPV for recall decreased substantially (from 68% to 25%), a result driven by the larger denominator of recalled cases.

Negative predictive value (NPV) remained uniformly high across all studies in which it was calculable, consistently exceeding 92%. This finding underscores ABUS’s value as a rule-out test. Inter-reader and inter-modality agreement, measured using Cohen’s κ, ranged from 0.48 for recall versus no-recall decisions to 0.85 for benign versus malignant classifications. Most studies reported substantial agreement, with κ values at or above 0.6.

Workflow metrics also demonstrated practical advantages. Technologist-performed ABUS acquisition required 3–5 min per breast, with the prone Sofia system achieving acquisition times as short as 35 s. Radiologist interpretation times averaged 3–5 min per exam, which was shorter than real-time HHUS (5–20 min) and comparable to the time required to read digital mammography.

### 3.4. Sensitivity Analysis

Meta-analysis was not feasible for low-risk studies because only two qualified, and they differed substantially in methodology, population, index test, and setting and therefore we refrain from presenting a “best-evidence” pooled estimate.

### 3.5. Publication Bias

Funnel plots for sensitivity stratified by modality did not show marked asymmetry, and Egger’s tests were non-significant for both ABUS (*p* = 0.79) and HHUS (*p* = 0.81), suggesting no clear evidence of publication bias.

Funnel plots for specificity by modality did not display marked asymmetry, and Egger’s tests were non-significant for both ABUS (*p* = 0.95) and HHUS (*p* = 0.77), indicating no clear evidence of publication bias.

### 3.6. Certainty/Confidence in the Evidence

Overall, the certainty of the evidence ranged from moderate to very low across outcomes. For cancer detection and sensitivity, certainty was mainly downgraded for risk of bias and inconsistency due to variability in study design and populations, as well as imprecision of some pooled estimates. Specificity and recall outcomes were generally rated as low certainty because of heterogeneity and concerns about indirectness, particularly in enriched or recall cohorts. Potential publication bias further reduced confidence for some comparisons, so the findings should be interpreted in light of these limitations.

## 4. Discussion

### 4.1. Summary of Results

Eighteen studies were included in this synthesis (approximately 20,000 screening or recall episodes). Overall, adding ABUS to mammography increased sensitivity and cancer detection in dense breasts at the expense of higher recall and modest specificity reductions.

Notably, only two studies were judged to have an overall low risk of bias, and they differed substantially in design and setting; therefore, we did not statistically pool them for the best evidence estimate.

Across the included studies, several design features appeared to be associated with differences in test accuracy. Population-based screening cohorts of women with dense breasts generally showed more modest gains in sensitivity with relatively small reductions in specificity, whereas enriched or recall populations and symptomatic outpatient cohorts more often demonstrated very high sensitivity at the expense of substantial specificity losses. Accuracy estimates also varied by setting and technology: multi-centre studies tended to yield slightly lower and less variable performance than single-centre studies, and some heterogeneity was observed between ABUS and automated breast volume scanners as well as between supine and prone acquisition systems, although patterns were not entirely consistent. Because the number of studies within each subgroup was limited and reporting of key design and patient characteristics was often incomplete, we did not undertake formal sensitivity or subgroup analyses. Instead, we provide a qualitative assessment of how these study characteristics may have influenced the pooled estimates, and these observations should therefore be interpreted as exploratory rather than definitive

### 4.2. Comparison with Prior Reviews

Our conclusions align with those of Gatta et al.’s systematic review and meta-analysis of screening in dense breasts, which found that adding 3D ABUS to mammography significantly increased cancer detection and reported higher overall sensitivity with combination imaging than with mammography alone, with some specificity penalty and higher recall. Their synthesis also quantified the absolute detection gains per 1000 screens in favour of adding ABUS [38].

For diagnostic accuracy against pathology, Zhang et al.’s meta-analysis comparing ABVS with HHUS (1376 patients) reported pooled sensitivity 0.93 vs. 0.86 vs. 0.82 (ABVS vs. HHUS), with no statistically significant difference on meta-regression supporting our finding that automated ultrasound is at least non-inferior to HHUS for lesion characterisation, while offering operational advantages [35].

### 4.3. Limitations and Strengths

This review applied a prespecified protocol, QUADAS-2 assessment, and state-of-the-art bivariate modelling of sensitivity and specificity.

Because 14/18 studies had a high risk of bias in patient selection and/or verification, the pooled estimates are hypothesis-generating and should not be interpreted as definitive population-screening performance.

Statistically, although we pre-specified random-effects modelling, substantial heterogeneity remained (especially for specificity), which limited the precision of the pooled summary points.

A key limitation of our assessment of publication bias is the small number of studies available for several of the pairwise comparisons. We cannot exclude the possibility that relevant unpublished or selectively reported studies were missed, and any residual publication bias could have influenced our pooled estimates.

An additional limitation is the substantial heterogeneity between studies and the correlation of study design and population with observed accuracy, which together limit the generalisability of our pooled estimates. These interrelated factors could not be fully disentangled, and our pooled results should therefore be interpreted as indicative of overall patterns rather than precise predictions for any screening programme or clinical setting.

### 4.4. Implications for Practice

Although ABUS and HHUS both show favourable diagnostic performance in dense breasts, real-world adoption of these modalities as routine supplemental screening remains limited. Our review suggests that ABUS could help address some operational barriers to HHUS (notably operator dependence and scan time) and is therefore well suited to high-volume settings. However, the observed increase in recall, concerns about overdiagnosis, the need for dedicated equipment and technologist training, and the predominance of high-risk-of-bias studies likely contribute to cautious uptake. In the absence of robust evidence on interval cancer rates, cost-effectiveness and patient-centred outcomes, many programmes have chosen to restrict supplemental ultrasound to selected high-risk or symptomatic populations rather than implementing it universally for all women with dense breasts. There is some evidence of gradual growth in the use of ABUS and HHUS in opportunistic screening and in regions with dedicated dense-breast clinics. However, our findings highlight that implementation has not yet become widespread in organised population screening programmes, largely because the balance between additional cancer detection and increased recall remains uncertain at a programme level.

### 4.5. Implications for Future Research

Population-based trials compared mammography ± ABUS (and ABUS vs. HHUS as a supplemental test) with standardised endpoints (interval cancer rates, PPV, recalls, and mortality surrogates) in the BI-RADS C/D cohorts. Methodological rigour with prospective consecutive recruitment, blinded independent readings, and uniform reference standards (histology for BI-RADS 4/5 plus ≥18- to 24-month follow-up for negatives) to mitigate the selection and verification biases observed in the current literature. Workflow and implementation with reader learning curves, quality assurance, and cost-effectiveness in high-volume screening settings where early signals favour throughput automation. Subgroup analyses were stratified by breast density, age, prior imaging, and ABUS platform (supine vs. prone, ABUS vs. ABVS), and incorporated patient-reported outcomes (anxiety and comfort) and equity of access.

## 5. Conclusions

ABUS appears to be a practical and scalable adjunct to mammography for women with dense breasts, increasing cancer detection at the cost of higher recall rates, with the magnitude of this trade-off varying by population and study design. The comparative accuracy of HHUS was broadly non-inferior, suggesting that local resources and workflows may guide the choice between modalities. Implementation in routine practice should be accompanied by structured reader training and ongoing quality assurance, including monitoring of recall rates and PPV. To strengthen the evidence base and improve generalisability, high-quality population-based screening trials and more standardised reporting of study methods and outcomes are still needed.

## Data Availability

The original contributions presented in this study are included in the article and Supplementary Material. Further inquiries can be directed to the corresponding author.

## Supplementary Materials

The following supporting information can be downloaded at: https://www.mdpi.com/article/10.3390/jimaging12010003/s1, Table S1: Characteristics of included studies; Table S2: Index tests, comparators, and reference standards; Table S3: Risk of bias assessment (QUADAS-2 domains); Table S4: Summary of main outcomes and funding by study.

## Abbreviations

The following abbreviations are used in this manuscript:

ABUS	Automated breast ultrasound
ABVS	Automated breast volume scanner
ACR	American College of Radiology
AUC	Area under the (receiver operating characteristic) curve
BI-RADS	Breast Imaging Reporting and Data System
CDR	Cancer detection rate
CI	Confidence interval
DBT	Digital breast tomosynthesis
FFDM	Full-field digital mammography
FDA	(US) Food and Drug Administration
HHUS	Handheld (whole-breast) ultrasound
HSROC	Hierarchical summary receiver operating characteristic
MRI	Magnetic resonance imaging
NPV	Negative predictive value
PPV	Positive predictive value
PPV1	Positive predictive value for recall
PPV3	Positive predictive value for biopsy
PRISMA	Preferred Reporting Items for Systematic Reviews and Meta-Analyses
QUADAS-2	Quality Assessment of Diagnostic Accuracy Studies 2
UST	Ultrasound tomography
3D	Three-dimensional
