# Supplementary material for: Adjunct Automated Breast Ultrasound in Mammographic Screening: A Systematic Review and Meta-Analysis
Source: J Imaging. 2025 Dec 22;12(1):3. doi: 10.3390/jimaging12010003 (PMC12843147; doi:10.3390/jimaging12010003)
Supplement: Supplementary file 1 [file jimaging-12-00003-s001.zip › jimaging-3978550-supplementary.pdf]

Table S1. Characteristics of included studies (N = 18).

No.	Study	Sample Size	Study Design	Country	Setting & Population	Inclusion Criteria	Exclusion Criteria	Age (Years)
1	Ahmed, 2022	62 women	Prospective diagnostic-accuracy (ABUS vs HHUS)	Iraq	Tertiary breast-imaging service; diagnostic work-up of women recalled from mammography with dense breasts	Diagnostic mammogram with ACR density C/D	ACR density A/B; refusal of ABUS; recent breast intervention	Mean 46.7 ± 5.9
2	Choi, 2014	5,566 women	Retrospective cohort with random allocation (ABVS vs HHUS)	Republic of Korea	Secondary-care radiology service; supplemental screening / benign follow-up	Asymptomatic women with BI-RADS 1–3 benign findings on prior HHUS within 6–12 months	Axillary or other non-breast imaging abnormalities; <12-month reference follow-up	Mean 47 ± 9 (19–82)
3	den Dekker, 2024	501 women	Prospective diagnostic-accuracy	Netherlands	Diagnostic work-up after population-based screening recall (BI-RADS 0) in three hospitals	Women 50–75 y recalled with BI-RADS 0 mammogram referred to participating hospitals	Non-consent; anxiety, time constraints, physical impairment; unavailability of ABUS	Median 55 (IQR 51–64)
4	Gatta, 2021	1,165 women	Prospective screening cohort; FFDM vs FFDM + prone ABUS	Italy	Hospital-based supplemental screening clinic for dense-breast women	Women 40–75 y, BI-RADS density 3/4, asymptomatic, no implants, no recent breast surgery/biopsy, no cancer history	Pregnancy; breast symptoms; surgery or biopsy within 12 months	Mean 47 y (ACR-C), 50 y (ACR-D)
5	Gouda, 2024	500 women	Retrospective diagnostic-accuracy (FFDM vs FFDM + ABUS)	Egypt	Hospital-based supplemental screening for dense breasts	Women 40–60 y, BI-RADS density C/D undergoing FFDM screening	BI-RADS 3 examinations (short-term follow-up)	Mean 48.45 ± 7.06 (40–60)
6	Guldogan, 2022	592 women	Prospective paired diagnostic-accuracy (ABUS vs HHUS)	Turkey	Hospital breast-imaging service; mixed screening/diagnostic evaluation of dense-breast women	ACR C/D on mammography or women <40 y without mammography	Prior breast cancer, implants, pregnancy/lactation, inflammatory skin disease, refusal, inadequate follow-up	Not reported
7	Hellgren, 2017	113 women	Prospective paired diagnostic-accuracy (ABVS vs HHUS)	Sweden	Recall assessment clinic within population-based screening	Women recalled from screening mammography for suspicious findings and able to undergo HHUS	Attendance on days without ABVS-trained staff or scanner time	Mean 55.6
8	Jia, 2020	937 women	Prospective multicentre cross-sectional	China	Five tertiary hospitals; diagnostic imaging adjunct to mammography	Women 40–69 y with mammographically dense breasts (BI-RADS c/d) undergoing MG, ABUS and HHUS	Age 30–39 y; missing density; non-dense breasts; prior BI-RADS 0	Mean 49.1 ± 6.8
9	Mohamed, 2020	20 women (20 breasts)	Prospective cross-sectional diagnostic-accuracy (MG, ABUS, HHUS)	Egypt	Tertiary breast-imaging clinic; symptomatic women from surgical out-patient clinic	Women 35–55 y with dense breasts (ACR C/D) consenting to additional imaging	Fatty breasts (ACR A/B)	Mean 43.4 ± 6.2 (35–55)
10	Mohammed, 2018	25 women	Prospective single-arm accuracy (ABUS vs MG; HHUS baseline)	Egypt	Supplemental screening / diagnostic clinic for dense breasts	Women presenting for screening, especially with dense breasts, or requiring supplementary US after positive mammography	None stated	Mean 43.4 ± 9.08
11	Niu, 2019	398 women; 599 masses	Prospective diagnostic-accuracy (ABUS vs HHUS)	China	Out-patient diagnostic assessment at first breast evaluation	Women with ≥1 solid or complex cystic mass and first clinic visit for breast pain, self-palpated concern or routine check	Suspicious clinical signs (e.g., bloody discharge, skin changes), previous cancer or augmentation, chest-wall deformity	Mean 39 (29–64)

12	Pawlak, 2023	297 women	Retrospective diagnostic-accuracy (ABUS vs FFDM; CEM subset)	Poland	Hospital breast-diagnostics unit; opportunistic screening/diagnostic work-up	Women undergoing screening FFDM who also had ABUS (with CEM if focal lesion)	Not explicitly reported	Mean 53.6
13	Philadelpho, 2021	440 women	Prospective cross-sectional (ABUS vs HHUS)	Brazil	Out-patient supplemental screening clinic for dense-breast women	Asymptomatic women with screening MG showing BI-RADS C/D density consenting to same-day HHUS + ABUS	Breast surgery or radiotherapy in prior 12 months; breast implants	Median 48
14	Tutar, 2020	340 women (of 345 eligible)	Prospective single-centre diagnostic-accuracy (ABVS vs HHUS)	Turkey	Secondary-care clinic; supplemental screening after negative MG	Women 35–67 y, ACR density B–D, negative 2-view MG, asymptomatic, willing to have both US exams	High-risk status; suspicious clinical findings; prior breast cancer; poor-quality/aborted ABVS	Median 49 (35–67)
15	Wilczek, 2016	1,668 women	Prospective paired-comparison (FFDSM vs FFDSM + 3D ABUS)	Sweden	High-volume population screening centre	Asymptomatic screening invitees ≥40 y with ACR 3/4 density	Pregnancy/breast-feeding; breast surgery or any cancer diagnosis/treatment within 12 months	40–74 (mean/SD not reported)
16	Xin, 2021	1,947 women	Prospective multicentre non-inferiority (HHUS vs ABUS ± MG)	China	Out-patient diagnostic breast clinics in tertiary hospitals	Women 30–69 y visiting for breast examination, no visible cancer signs, consenting to imaging	Pregnancy/lactation; prior breast surgery or augmentation; biopsy or cancer treatment within 12 months	Overall 45.4 ± 9.8
17	Zhang, 2018	1,973 women	Prospective multicentre non-inferiority (ABUS vs HHUS; ABUS vs MG)	China	Diagnostic evaluation of symptomatic out-patients at tertiary centres	Women 30–69 y attending breast clinics, no prior breast cancer/therapy, consenting to HHUS + ABUS (plus MG if 40–69 y)	Pregnancy/lactation; prior surgery, biopsy or cancer treatment; augmentation; age <30 or ≥70 y	Overall 45.4 ± 9.7
18	Zhang, 2019	385 women; 594 breast units	Prospective comparative diagnostic-accuracy	China	Tertiary hospital breast clinic; diagnostic/clinical assessment	Women ≥40 y attending for breast assessment and consenting to imaging	Pregnancy/breast-feeding or planned pregnancy; other malignancy; prior breast surgery/biopsy or mastectomy; augmentation	Mean 50.96 ± 7.05 (40–69)

Table S2. Index tests, comparators, and reference standards.

No.	Study	Readers	Index Test vs. Comparator	Imaging Protocol (ABUS and Comparator)	ABUS Platform	Reference Standard
1	Ahmed, 2022	Not reported	ABUS vs HHUS for lesion detection and BI-RADS	HHUS: GE LOGIQ S8, 4–15 MHz; bilateral radial/anti-radial + axilla (~20 min). ABUS: ≥3 supine sweeps/breast	Siemens Acuson S2000 ABVS; volumetric 3D acquisition with axial/sagittal/coronal review	Core-needle biopsy for all BI-RADS 4–5 and a random subset of BI-RADS 3 lesions
2	Choi, 2014	Five board-certified breast radiologists (1–11 y)	ABVS vs HHUS (BI-RADS-based)	ABVS: 3 automated 3D scans/breast (0.5 mm slices). HHUS: two perpendicular 2D passes/breast	ACUSON S2000 (Siemens) wide-footprint 5–14 MHz transducer	Histopathology for positives plus ≥12-month imaging and clinical follow-up for negatives
3	den Dekker, 2024	Fifteen breast radiologists (1.5–29 y)	ABUS added to DBT + HHUS vs DBT + HHUS alone	HHUS: 14L5 probe. ABUS: 3–5 volumetric scans/breast (AP, LAT, MED ± SUP/INF), 0.5 mm slices	Invenia™ ABUS (GE Healthcare); 6–15 MHz wide transducer; technician-operated	Histopathology for BI-RADS 4/5; ≥6-month follow-up for BI-RADS 3 without biopsy
4	Gatta, 2021	Three breast radiologists (10–20 y)	FFDM alone vs FFDM + prone ABUS	FFDM: 2–3 views as per protocol. Prone ABUS: single 360° sweep/breast with multiplanar reconstructions	Sofia™ prone ABUS system (Hitachi Arietta/Sofia)	Image-guided biopsy for BI-RADS 4/5; benign/probably benign monitored with 24-month follow-up
5	Gouda, 2024	Two breast radiologists (15 y)	FFDM alone vs FFDM + ABUS (HHUS for biopsy guidance only)	FFDM: 2-view digital. ABUS: ≥3 sweeps (AP, medial, lateral; extra for large breasts). HHUS: LOGIQ 9, 7–11 MHz	GE Invenia™ ABUS 2.0; supine scanning, 10–20 min acquisition	US-guided core biopsy for BI-RADS 4/5; typical benign lesions classified by imaging only
6	Guldogan, 2022	Three breast radiologists (2–20 y)	ABUS vs HHUS	ABUS: ≥3 sweeps/breast (AP, lateral, medial; extra for large breasts). HHUS: LOGIQ S8, 6–15 MHz with radial/anti-radial passes and Doppler	Invenia™ ABUS (GE Healthcare), technician-acquired, multiplanar review	US-guided core biopsy for BI-RADS 4/5 (40 lesions); benign lesions followed
7	Hellgren, 2017	Five radiologists (4–28 y); one radiographer acquired ABVS	ABVS vs HHUS	ABVS: ≥3 sweeps/breast (AP, lateral, medial; 0.5 mm slices). HHUS: Philips iU22, 17–5 or 12–5 MHz with radial/anti-radial scans incl. axilla	Siemens Acuson S2000 ABVS workstation with axial/sagittal/coronal review	Core-needle or FNA biopsy for BI-RADS ≥3; BI-RADS 1–3 followed to next screening (12–24 months)
8	Jia, 2020	Radiologists blinded across modalities; exact number/experience not reported	MG alone; MG + ABUS; MG + HHUS; standalone ABUS and HHUS in MG-negative subset	Bilateral 2-view digital MG plus whole-breast ABUS (3 views/breast) and HHUS	Commercial ABUS platform (model not specified); 3 standard sweeps with coronal reconstruction	Core biopsy for BI-RADS 4–5 or BI-RADS 3 with abnormal MRI; 10% of negatives verified by MRI/biopsy
9	Mohamed, 2020	HHUS performed and read by one radiologist; ABUS read by study radiologist	MG vs ABUS vs HHUS	MG: standard 2-view digital. ABUS: ≥3 volumetric sweeps/breast (1.7 mm slices). HHUS: 7–11 MHz with radial/anti-radial and Doppler incl. axilla	Invenia™ ABUS (GE Healthcare); automated 3D acquisition with workstation review	Histopathology (core biopsy or FNA) for all patients within 7–15 days
10	Mohammed, 2018	Not reported	ABUS vs digital MG (HHUS used only for triage)	MG: 2-view FFDM. ABUS: ≥3 sweeps/breast with optional upper-quadrant/axillary views. HHUS: handheld 7–13 MHz probe	GE Invenia ABUS; automated acquisition (~7 min bilateral) with axial/coronal reconstructions	BI-RADS categorisation as outcome; pathology confirmation not systematically reported
11	Niu, 2019	Four sonographers; 2 HHUS operators (>7 y) and 2 ABUS readers (>5 y)	HHUS vs ABUS for classification of breast masses	HHUS: 6–18 MHz linear probe. ABUS: ≥3 3D volumes/breast (supine) with multiplanar reconstructions	U-Systems somo·v ABUS; automated 6–14 MHz transducer, dedicated workstation	Histopathology for 378 masses plus ≥1-year imaging follow-up for BI-RADS 2–3 lesions
12	Pawlak, 2023	Two breast radiologists (5–30 y)	FFDM alone; ABUS alone; CEM subset; FFDM + ABUS	FFDM: two-view digital. ABUS: ≥3 sweeps/breast (AP, lateral, medial; extra if needed). CEM: dual-energy CC and MLO	Invenia™ ABUS (GE Healthcare), technician-acquired with multiplanar review	Core or vacuum biopsy for BI-RADS 4/5; dedicated breast pathology for all sampled lesions

13	Philadelpho, 2021	30 HHUS operators (13 breast, 17 general); 6 breast radiologists read ABUS	HHUS vs ABUS	ABUS: 3–4 sweeps/breast (0.5 mm slices; 6–8 views typical). HHUS: 7–14 MHz linear probes with radial/anti-radial and axillary assessment	Invenia™ ABUS 2.0; supine scanning with workstation multiplanar review	Core biopsy for BI-RADS 4 lesions; BI-RADS 1–3 managed per routine without systematic follow-up
14	Tutar, 2020	HHUS by 2 senior breast radiologists; ABVS read in consensus; 4 technologists acquired ABVS	ABVS vs HHUS	ABVS: 3 axial 3D sweeps/breast (extra views in a minority). HHUS: bilateral scans with 14L5/9L4 probes including axilla	Siemens ACUSON S2000 ABVS; 5–14 MHz, ~60 s per sweep	US-guided core biopsy for BI-RADS 4/5; ABVS-only lesions and BI-RADS 3 followed for 36–48 months
15	Wilczek, 2016	Two senior breast radiologists	FFDSM alone vs FFDSM + 3D ABUS	FFDSM: 2-view FFDM. ABUS: AP, lateral and medial sweeps/breast with 0.5 mm slices	U-Systems prototype (later Invenia™); technician-acquired, multiplanar reconstruction	Standard screening work-up with MG ± HHUS and biopsy for BI-RADS 4/5; interval cancers identified at 18–24 months
16	Xin, 2021	10 HHUS radiologists; 5 ABUS readers; 10 mammographers; 5 MRI radiologists (all ≥5 y)	ABUS vs HHUS; MG only in women 40–69 y	ABUS: three 3D sweeps/breast (~300 slices/view, 0.5 mm). HHUS: various platforms with 5–14 MHz probes; MG: standard 2-view digital	Invenia™ ABUS; automated supine scanning with "thick-slice" coronal navigation	Histopathology for BI-RADS 4/5; MRI for BI-RADS 3 and 10% BI-RADS 1–2; clinical follow-up for remaining negatives
17	Zhang, 2018	10 HHUS radiologists, 5 ABUS readers, 10 mammographers (≥5 y)	ABUS alone; comparisons with HHUS (all ages) and MG (40–69 y)	HHUS: multi-vendor 5–14 MHz probes. ABUS: three 3D sweeps/breast (~300 slices, 0.5 mm). MG: standard 2-view digital	Invenia™ ABUS; ~60 s per sweep, 3–4 min per breast with coronal "survey mode"	Core/surgical pathology for BI-RADS 4–5; MRI for BI-RADS 3 and 10% BI-RADS 1–2; clinical follow-up for others
18	Zhang, 2019	Two HHUS readers; two ABUS readers; two MG radiologists (all >7 y)	HHUS vs ABUS vs MG	HHUS, ABUS and MG all performed; MG with CC and MLO views; ABUS with ≥3 standard volumetric views/breast and additional views for large breasts	U-Systems somo·v ABUS; automated 6–14 MHz transducer with coronal reconstruction	Pathology for BI-RADS 4–5 or lesions with >20% growth; MRI for BI-RADS 3 and 20% of BI-RADS 1–2; otherwise imaging follow-up

Table S3. Risk of bias assessment (QUADAS-2 domains).

No.	Study	Patient selection	Index test	Reference standard	Flow and timing
1	Ahmed, 2022	**High** – small, symptomatic convenience cohort of women with dense diagnostic MG at a tertiary oncology hospital	**Unclear** – blinding of ABUS readers to HHUS/clinical data not reported	**High** – only BI-RADS 4/5 and some BI-RADS 3 biopsied; others assumed benign	**Low** – HHUS and ABUS same visit; all participants entered verification pathway
2	Choi, 2014	**High** – retrospective chart review of two non-consecutive cohorts (ABVS vs HHUS)	**Unclear** – same radiologists read both modalities; blinding not stated	**Unclear** – biopsies for BI-RADS 4/5; BI-RADS 1–3 verified only by ≥12-month follow-up	**High** – mixed verification methods and slightly different follow-up intervals
3	den Dekker, 2024	**High** – only 47% of BI-RADS 0 recalls enrolled; exclusions driven by logistics and refusal	**High** – ABUS read after DBT + HHUS by same radiologist (incorporation bias)	**Low** – histology for BI-RADS 4/5; structured follow-up for BI-RADS 3; minimal loss	**Unclear** – different reference procedures across BI-RADS categories
4	Gatta, 2021	**Low** – consecutive asymptomatic dense-breast screening invitees with standard exclusions	**Low** – ABUS interpreted by a blinded reader after FFDM; no access to final work-up	**Low** – biopsies for positives; registry-based interval-cancer ascertainment for negatives	**Low** – same-day index tests; uniform verification; no reported losses
5	Gouda, 2024	**High** – retrospective single-year dense-breast screens; BI-RADS 3 and non–40–60 y excluded	**High** – ABUS read by same radiologists after FFDM, without reported blinding	**High** – only BI-RADS 4/5 biopsied; many negatives assumed benign	**Low** – FFDM and ABUS same day; no losses reported
6	Guldogan, 2022	**High** – randomly/proportionally selected dense-breast out-patients; enriched diagnostic spectrum	**Unclear** – HHUS performed first; blinding of ABUS readers to HHUS/clinical data not specified	**High** – only BI-RADS 4/5 biopsied; BI-RADS 1–3 treated as benign after follow-up	**Low** – both tests same visit; all breasts had histology or ≥20-month follow-up
7	Hellgren, 2017	**High** – convenience sample of recalls limited by staff/scanner availability	**Unclear** – ABVS interpreted by same radiologists after HHUS; blinding not stated	**Low** – biopsies for BI-RADS 4/5; BI-RADS 1–3 followed to next screening	**High** – differential verification and operational exclusions of recalls
8	Jia, 2020	**High** – convenience sample of dense-breast out-patients at tertiary centres, not screening invitees	**Unclear** – blinding of ABUS readers to HHUS/clinical data not reported	**Unclear** – biopsies for positives; short follow-up and incomplete MRI in negatives	**Low** – same-visit imaging; uniform verification within BI-RADS strata; minimal loss
9	Mohamed, 2020	**High** – very small symptomatic cohort of women consenting to ABUS	**Unclear** – HHUS performed first by same radiologist; ABUS blinding not stated	**Low** – all lesions underwent biopsy or FNA; full pathological verification	**Low** – MG, HHUS and ABUS same visit; no losses to follow-up
10	Mohammed, 2018	**High** – small, non-consecutive pilot of self-selected women with dense breasts	**High** – same radiologist performed MG and HHUS and then reviewed ABUS	**Unclear** – reference standard not clearly described for all positives/negatives	**Low** – all tests completed at one visit; no participants excluded
11	Niu, 2019	**High** – randomly/proportionally selected symptomatic out-patients; not screening cohort	**Unclear** – HHUS performed first; ABUS read later; blinding not reported	**High** – biopsies for BI-RADS 4/5; limited MRI and follow-up in BI-RADS 1–3	**Low** – sequential HHUS and ABUS same visit; acceptable test–reference interval
12	Pawlak, 2023	**High** – retrospective single-centre cohort enriched for ACR C/D; not consecutive screening sample	**Unclear** – two radiologists read all modalities; blinding to verification not explicit	**High** – only BI-RADS 4/5 biopsied; negatives verified mainly by routine follow-up	**Low** – all participants received each index test same day; no differential loss reported
13	Philadelpho, 2021	**Low** – consecutive asymptomatic dense-breast screening cohort with minimal exclusions	**Low** – HHUS and ABUS read by separate readers, blinded to each other	**High** – only BI-RADS 4/5 biopsied; no systematic verification for BI-RADS 1–3	**High** – verification differed by BI-RADS category; no follow-up for BI-RADS 3
14	Tutar, 2020	**Low** – prospective consecutive dense-breast cohort with negative MG invited for both tests	**Unclear** – same radiologists interpreted HHUS and ABVS; blinding not clearly stated	**Low** – positive findings biopsied; negatives followed for ≥36 months	**Low** – both tests same visit; complete follow-up with no meaningful attrition
15	Wilczek, 2016	**Low** – consecutive asymptomatic screening invitees with ACR 3/4; minimal protocol violations	**High** – first reader interpreted FFDSM and then ABUS (non-independent reading)	**Low** – standard work-up with biopsy for BI-RADS 4/5 and interval-cancer capture	**Low** – FFDSM and ABUS same visit; uniform verification; very low exclusion rate
16	Xin, 2021	**High** – hospital-based symptomatic attendees; not population screening	**Low** – HHUS, ABUS and MG interpreted independently with explicit blinding	**High** – biopsies for BI-RADS 4/5; partial MRI sampling and unverified negatives	**Unclear** – verification pathway varied by BI-RADS and age; MG not performed in all
17	Zhang, 2018	**High** – proportionally selected symptomatic out-patients; broad age exclusions; many decliners	**High** – highest BI-RADS score across ABUS, HHUS and MG used for work-up (non-independent tests)	**High** – biopsies for positives; MRI for BI-RADS 3; many BI-RADS 1–2 without verification	**High** – large post-enrolment exclusions and differential verification by BI-RADS

18	Zhang, 2019	**High** – enriched clinic cohort with multiple post-enrolment exclusions	**Low** – ABUS, HHUS and MG read by separate blinded experienced readers	**High** – composite reference (pathology or imaging follow-up); many negatives unbiopsied	**High** – substantial exclusions and differing follow-up schedules across BI-RADS strata

Table S4. Summary of main outcomes and funding by study.

No.	Study	Main Results (ABUS/ABVS vs. Comparator)	Funding
1	Ahmed 2022	ABUS showed higher sensitivity (93.8% vs 87.5%) and specificity (70.6% vs 58.8%) than HHUS, with better accuracy (81.8% vs 72.7%). Mass-detection agreement between ABUS and HHUS was substantial (κ = 0.79); BI-RADS agreement was also substantial (κ = 0.72).	No specific funding reported.
2	Choi 2014	Compared ABVS with HHUS. Recall was lower with ABVS (2.57 vs 3.57 per 1,000; p = 0.048), with similar cancer detection (3.8 vs 2.7 per 1,000; p = 0.503). Accuracy was slightly higher (97.7% vs 96.5%; p = 0.018) with modest gains in sensitivity and specificity and similar PPV/NPV.	Funding not reported.
3	den Dekker 2024	ABUS alone had sensitivity 72.1% and specificity 84.4% (PPV 29.2%, NPV 97.1%), missing 12/43 cancers (27.9%). DBT + HHUS achieved 100% sensitivity with lower specificity (71.4%) and PPV 23.8%, but NPV 100%.	Supported by research funding from General Electric (GE); funder had no role in the study conduct.
4	Gatta 2021	Adding prone ABUS to FFDM doubled the cancer detection rate (3.4 → 6.8 per 1,000; p < 0.007) and increased sensitivity (58.8% → 93.5%; p < 0.001) at the cost of reduced specificity (94% → 87%; p < 0.001), higher recall (14.5 → 26.6 per 1,000) and biopsy rates. Inter-reader agreement for ABUS was substantial (Fleiss κ = 0.72).	Funded by the Italian Ministry of Health.
5	Gouda 2024	Integration of ABUS reduced recalls (202/500 → 131/500; p < 0.001) and slightly decreased biopsy numbers (141 → 121) while increasing cancers detected (71 → 111; 22.2% malignant). PPV for biopsy improved from 74.5% to 83.5%; overall agreement between modalities was moderate (κ = 0.51).	No external grants reported.
6	Guldogan 2022	HHUS detected more lesions than ABUS (1,827 vs 1,275), but lesion-level agreement was moderate to substantial (κ ≈ 0.57–0.66). For 11 cancers, sensitivity and NPV were 100% for both. ABUS had lower specificity and accuracy than HHUS (82.4% vs 91.4% and 82.8% vs 91.6%) with higher recall (19% vs 10.3%), but higher PPV for recalled lesions (PPV2 38.4% vs 21.2%).	No funding reported.
7	Hellgren 2017	In breasts with mammographic suspicion, HHUS and ABVS had identical sensitivity (88%; 22/25), with slightly higher specificity for HHUS (93.5% vs 89.2%, p = 0.29). In contralateral negative breasts, both achieved 100% sensitivity, while specificity was higher for HHUS (100% vs 94.1%, p = 0.03).	No funding reported.
8	Jia 2020	In dense breasts, MG + ABUS and MG + HHUS achieved very high sensitivity (both 99.1%) with slightly higher specificity for ABUS (86.9% vs 84.9%; AUC 0.93 vs 0.92). In the MG-negative subgroup, ABUS and HHUS had similar sensitivity (93.6%), with ABUS showing higher specificity (96.1% vs 94.0%) and PPV (53.7% vs 42.7%). Incremental cancer detection was 42.8 per 1,000; agreement between ABUS and HHUS was almost perfect (κ = 0.85).	Supported by GE Healthcare (Grant CH-EPI-027) and the Chinese Academy of Medical Sciences Innovation Fund for Medical Sciences.
9	Mohamed 2020	Per-patient accuracy was highest for HHUS (sensitivity 80%, specificity 90%, accuracy 85%). ABUS improved sensitivity over mammography alone (60% vs 30%) but had lower specificity (70% vs 100%) and similar accuracy (65%). Authors concluded that ABUS enhances cancer yield in dense breasts, but HHUS remains the most accurate modality.	No funding reported.
10	Mohammed 2018	ABUS achieved sensitivity 100%, specificity 62.5%, PPV 85%, NPV 100% and accuracy 88%. Mammography had a 68% overall positive rate but missed three malignant BI-RADS 4/5 lesions that were ABUS-positive. Mean BI-RADS scores were slightly higher with ABUS than mammography, although this difference was not statistically significant.	No external grants reported.
11	Niu 2019	ABUS demonstrated higher sensitivity than HHUS (92.2% vs 81.6%) with slightly lower specificity (77.6% vs 80.0%); overall accuracy was similar (~80%). PPV and NPV were comparable, and the AUC was significantly higher for ABUS (0.85 vs 0.81; p = 0.041).	Funding not reported.
12	Pawlak 2023	In dense breasts (ACR C/D), ABUS sensitivity was 78.1% with specificity 40.0%; FFDM had higher sensitivity (85.4%) but very low specificity (13.3%), while CEM showed the highest sensitivity (92.7%) but also low specificity. Combining FFDM + ABUS	No external funding declared.

		achieved 100% sensitivity with specificity 6.7% (AUC 0.921). In non-dense breasts, patterns were similar, with ABUS and FFDM both highly sensitive but poorly specific.	
13	Philadelpho 2021	ABUS detected more cancers than HHUS (4.5 vs 2.3 per 1,000) with slightly shorter reading time (4 min 25 s vs 5 min 03 s; $p < 0.001$). Overall concordance between ABUS and HHUS was high (80.9%).	Supported by Diagnósticos da América (DASA); the Invenia™ system was loaned by GE Healthcare.
14	Tutar 2020	After negative mammography, both ABVS and HHUS had 100% sensitivity and NPV. ABVS showed lower specificity and PPV (84.5% and 4.2%) and much higher recall (46/340) than HHUS (specificity 99.3%, PPV 50.0%, recall 4/340), though no interval cancers occurred at ≥36 months.	Funding not reported.
15	Wilczek 2016	Adding 3D ABUS to FFDSM increased cancer detection from 4.2 to 6.6 per 1,000 (Δ +2.4; $p < 0.001$), particularly for invasive cancers (Δ +2.1 per 1,000), but raised recall from 13.8 to 22.8 per 1,000 ($p = 0.004$). PPV for recall was similar (30.4% vs 28.9%).	Equipment loaned by U-Systems (Sunnyvale, CA); no other funding declared.
16	Xin 2021	Across all ages (n = 1,947), ABUS had sensitivity 91.8%, specificity 92.9% and accuracy 92.7%, while HHUS showed slightly higher sensitivity (94.7%) but lower specificity (89.4%) and accuracy (90.5%); ABUS specificity was significantly superior ($p < 0.001$). In women 40–69 years, ABUS had higher sensitivity than mammography (93.0% vs 86.0%; $p < 0.001$) with non-inferior specificity (92.9% vs 91.7%).	Investigator-initiated study sponsored by GE Healthcare.
17	Zhang 2018	In a similar multicentre cohort (n = 1,973), ABUS achieved sensitivity 91.8%, specificity 92.9% and accuracy 92.7%. HHUS again had higher sensitivity (94.7%) but lower specificity (89.4%) and accuracy (90.5%). In women aged 40–69 years, ABUS outperformed mammography for sensitivity (93.0% vs 86.0%) with comparable specificity, supporting non-inferiority to MG and near-equivalence to HHUS.	Supported by GE Healthcare (Grant CH-EPI-027); the funder had no role in analysis or reporting.
18	Zhang 2019	At the breast-unit level, HHUS had the highest sensitivity (97.3%) but lower specificity (89.8%), while ABUS balanced slightly lower sensitivity (90.7%) with higher specificity (92.5%) and the highest accuracy (92.3%). Mammography had the lowest sensitivity (84.0%). AUCs were high for all (HHUS 0.936, ABUS 0.916, MG 0.884; differences not statistically significant). ABUS reclassified ~45% of HHUS false positives correctly but missed 7 cancers.	Supported by the Health Commission of Zhejiang Province and the Science & Technology Program of Hangzhou.
